# The calcium connection: exploring the intricacies of calcium signaling in plant-microbe interactions

**DOI:** 10.3389/fpls.2023.1248648

**Published:** 2023-10-02

**Authors:** Neelam Prabha Negi, Geeta Prakash, Parul Narwal, Ruby Panwar, Deepak Kumar, Bharti Chaudhry, Anjana Rustagi

**Affiliations:** ^1^ University Institute of Biotechnology, Chandigarh University, Mohali, India; ^2^ Department of Botany, Gargi College, New Delhi, India; ^3^ Department of Botany, Institute of Science, Banaras Hindu University, Varanasi, Uttar Pradesh, India; ^4^ Department of Botany, Ramjas College, New Delhi, India

**Keywords:** Ca^2+^ signaling, plant microbe interaction, endophytes, biotic stress, omics, CRISPR

## Abstract

The process of plant immune response is orchestrated by intracellular signaling molecules. Since plants are devoid of a humoral system, they develop extensive mechanism of pathogen recognition, signal perception, and intricate cell signaling for their protection from biotic and abiotic stresses. The pathogenic attack induces calcium ion accumulation in the plant cells, resulting in calcium signatures that regulate the synthesis of proteins of defense system. These calcium signatures induct different calcium dependent proteins such as calmodulins (CaMs), calcineurin B-like proteins (CBLs), calcium-dependent protein kinases (CDPKs) and other signaling molecules to orchestrate the complex defense signaling. Using advanced biotechnological tools, the role of Ca^2+^ signaling during plant-microbe interactions and the role of CaM/CMLs and CDPKs in plant defense mechanism has been revealed to some extent. The Emerging perspectives on calcium signaling in plant-microbe interactions suggest that this complex interplay could be harnessed to improve plant resistance against pathogenic microbes. We present here an overview of current understanding in calcium signatures during plant-microbe interaction so as to imbibe a future direction of research.

## Introduction

Calcium ions (Ca^2+^) play an important role in various plant functions, like providing structural support, nutrition, and inducing stress responses. Besides these, Ca^2+^ functions as a secondary messenger in cell to cell signaling, and disruptions in its levels occur during biotic or abiotic stress responses (Ghosh et al., 2022). Under different stress conditions, there are spikes in Ca^2+^ concentration within the cell cytosol, known as “calcium signatures”. These Ca^2+^ spikes can be sensed by different calcium influx and efflux proteins, which maintain cytosolic Ca^2+^ homeostasis (Yadav et al., 2022). The calcium influx proteins facilitate the entry of calcium ions into the cell, initiating calcium signaling through the channels that allow Ca^2+^ to enter the cytosol. Conversely, calcium efflux proteins work to remove excess calcium ions from the cytosol, regulating the duration and magnitude of calcium signals and maintaining cellular calcium homeostasis. These proteins are critical components of calcium signaling pathways in plants, orchestrating various physiological processes encompassing growth, development, and the ability to mount responses against biotic and abiotic stresses (Ranty et al., 2016; Raina et al., 2021a; Verma et al., 2022).

**Figure 1:**
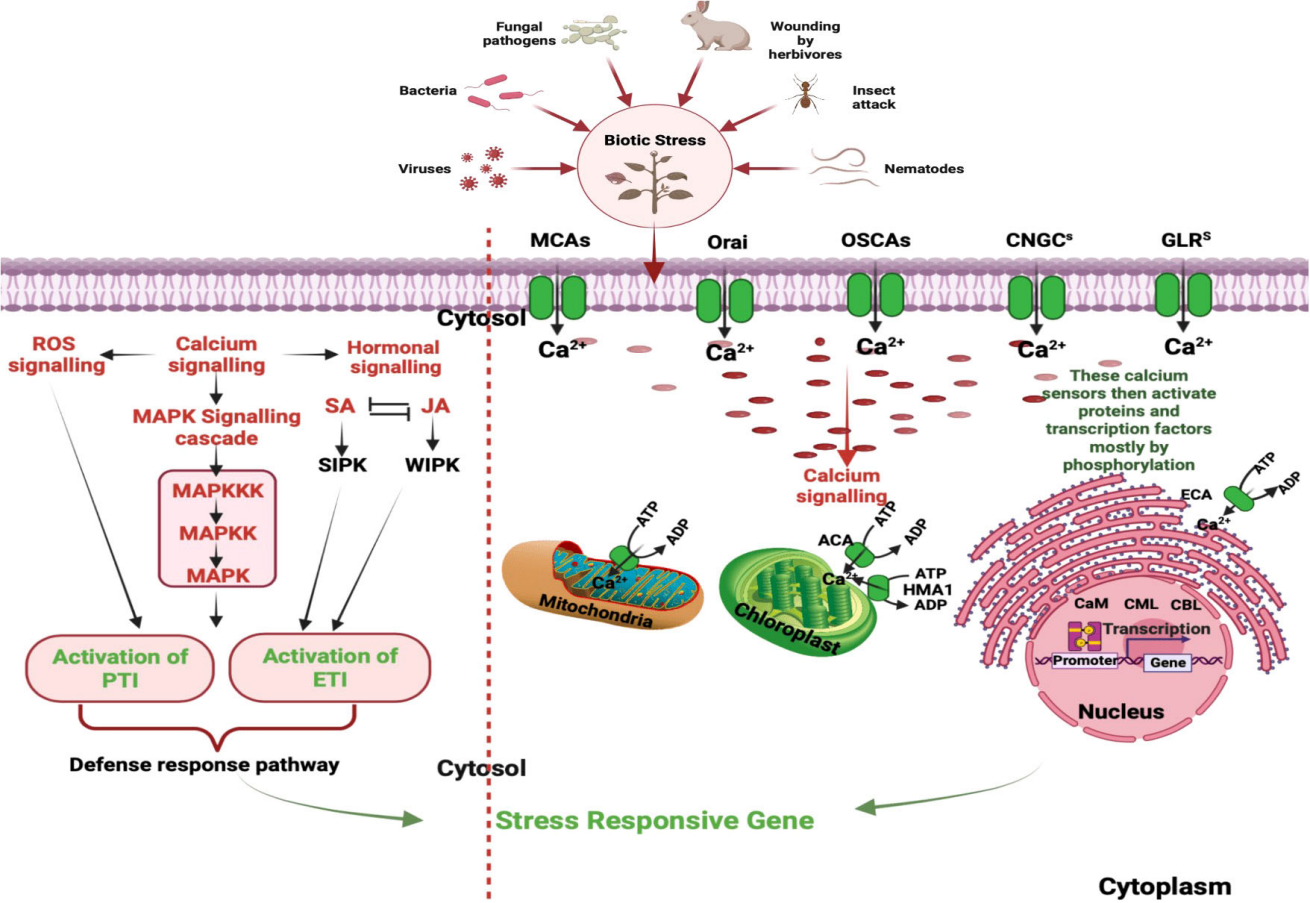
Stages in Ca^2+^ Signaling Pathways During Plant Interactions with Biotic stressors: This figure underscores the intricate network of calcium signaling components within plant cells. Calcium influx is mediated by diverse channels, including CNGCs, GLRs, TPCs, MCAs, and OSCAs. Meanwhile, calcium efflux is facilitated through systems like ACAs, ECAs, HMA1, MCUC, and CAX. Decoding is executed by specific protein families, encompassing CDPKs), calcineurin B-like protein kinases (CIPKs), calmodulin (CaM), and CaM-like proteins (CMLs). These processes play a pivotal role in perceiving and responding to a myriad of signals during plant interactions with both pathogenic and mutualistic organisms. Initial steps involve distinct cytosolic calcium spikes, referred to as calcium signatures, which encode the primary layer of specificity. Subsequent decoding of these calcium transients contributes to the second layer of specificity, ultimately culminating in the activation of target proteins within the defense cascade. Additionally, phytohormones such SA and JA, along with induced protein kinases and reactive oxygen species (ROS), contribute to the signaling cascade, culminating in intricate and coordinated plant defense reactions. [CNGCs: Cyclic Nucleotide-Gated Channels; GLRs: Ionotropic Glutamate Receptors; TPCs: Two-Pore Channel 1; MCAs: Mechanosensitive Protein Channels; CDPKs, Calcium-Dependent Protein Kinases; CBLs, Calcineurin B-Like Proteins; ACAs, Autoinhibited Ca^2+^-ATPases; ECAs, ER-Type Ca^2+^-ATPases; HMA1, P1-ATPase Heavy Metal Transporter 1; MCUC, Mitochondrial Calcium Uniporter Complex; CAX, Ca^2+^-Exchangers; SA, Salicylic Acid; JA, Jasmonic Acid; ROS, Reactive Oxygen Species.]

The dynamic control of intracellular calcium level relies on a delicate balance between extracellular Ca^2+^ entry and intracellular Ca^2+^ release from various subcellular compartments including mitochondria, endoplasmic reticulum and vacuoles (Resentini et al., 2021). The influx of extracellular Ca^2+^ is mediated by various calcium channel, such as plasma membrane localized calcium channels and the voltage-dependent calcium channels. These channels play crucial roles in regulating cellular Ca^2+^ levels and are essential for various physiological processes in plants (**Figure 1**, right panel). Additionally, the mobilization of Ca^2+^ from intracellular storage is actively facilitated by various signaling molecules such as inositol triphosphate (InsP_3_), cyclic adenosine diphosphate ribose (cADPR) and nicotinic acid adenine dinucleotide phosphate (NAADP) (Galione and Churchill, 2002). InsP3 is involved in the activation of Ca^2+^ release from the endoplasmic reticulum, while cADPR and NAADP participate in Ca^2+^ release from acidic organelles such as lysosomes and endosomes. In the InsP_3_ pathway, signaling molecules like hormones or environmental cues activate phospholipase C, which enzymatically hydrolyses phosphatidylinositol 4,5-biphosphate (PIP_2_) to produce diacylglycerol and InsP_3_. The InsP_3_ subsequently interacts with InsP_3_ receptors located on the endoplasmic reticulum, inducing the discharge of Ca^2+^ into the cytosol (Kudla et al., 2018). The overexpression of a phospholipase C gene resulted in elevated intracellular Ca^2+^ levels and increased resistance against *Pseudomonas syringae* (Meng et al., 2016). On the other hand, cADPR and NAADP promote the release of Ca^2+^ from acidic compartments such as vacuoles and lysosomes by activating ryanodine receptors (RyRs) and two-pore channels (TPCs), respectively (Berridge et al., 2000; Li et al., 2015). For example, the intracellular bacterial pathogen *Legionella pneumophila* can hijack host cellular machinery to promote the mobilization of Ca^2+^ from intracellular reservoirs, leading to increased replication within host cells (Swanson and Isberg, 1995; Machner and Isberg, 2006).

Furthermore, the calcium signaling pathway is intricately integrated with other signaling modules, hormonal signaling, and the production of reactive oxygen species (ROS) in plants. The CDPKs target different defense-responsive proteins and bring about resistance response (Singh et al., 2022). Additionally, calcium transport and storage in plants involve calcium-binding proteins, and transporters/pumps, that help chelate and buffer cytosolic Ca^2+^. Calcium flow occurs through the cell wall, vacuoles, apoplast and different cellular organelles (Mohanta et al., 2018). **Figure 1**, left panel represents a schematic diagram of calcium signaling components within plant cells.

During stress, changes in the water potential or ion concentration in the plant’s environment can activate signaling pathways that result in the influx of extracellular Ca^2+^ into the cytoplasm of plant cells (McAinsh and Pittman, 2009). This influx of calcium ions can trigger downstream signaling events that help the plant to adapt to the stressful conditions. For example, calcium influx can activate various CDPKs and CBPs, which can regulate gene expression and various cellular processes such as stomatal closure, ion transport, and osmotic adjustment (Kudla et al., 2010). The transport of Ca^2+^ across membranes is an essential aspect of calcium signaling in plants. Several classes of membrane transporters have been identified that play critical roles in regulating the intracellular calcium concentration [Ca^2+^]i. These include Ca^2+^-ATPases, Ca^2+^/H^+^ antiporters, Ca^2+^ channels, and Ca^2+^ permeable ion channels. Ca^2+^-ATPases are involved in active transport of Ca^2+^ out of the cytosol, contributing to the maintenance of low [Ca^2+^]i levels (Kudla et al., 2018). The Ca^2+^/H^+^ antiporters mediate the exchange of cytosolic Ca^2+^ and protons across the membranes, thus regulating the pH and Ca^2+^ homeostasis in different cellular compartments (White and Broadley, 2003). The Ca^2+^ channels facilitate the influx of extracellular Ca^2+^ into the cytosol and are activated by various stimuli, including voltage, mechanical stress, and ligands (Kudla et al., 2018). Calcium transporters have been investigated for their plausible role in plant responses to pathogenic stress and environmental cues. For instance, in *Arabidopsis*, the overexpression of a vacuolar Ca^2+^ATPase led to an increase in calcium accumulation in vacuoles and thereby increasing the tolerance to NaCl induced salt stress (Yamaguchi et al., 2013). In another study, the silencing of a plasma membrane Ca^2+^-ATPase reduced calcium transport and increased sensitivity to oxidative stress in *Nicotiana tabacum* (Crouzet et al., 2013). The compiled data on calcium transporters and their associated protein sensors have been meticulously presented in **Table 1**.

**Table 1:** List of calcium transporters and their protein sensors in different plants studied.

S.No.	Number of Calcium transporters	Sensor proteins	Category (Channel/Pump/Exchanger)	Plant species	Calcium Flux	Localization	References
1.	16	Cyclic nucleotide-gated channels (CNGCs)	Calcium channel	*Oryza sativa*	Calcium Influx	Plasma membrane, mitochondrial, nuclear, and vacuolar membrane	Nawaz et al., 2014
2.	20	CNGCs	Calcium channel	*Arabidopsis thaliana*	Calcium Influx	Plasma membrane, mitochondrial, nuclear, and vacuolar membrane	Schuurink et al., 1998
3.	1	Nucleotide-binding, leucine-rich repeat receptors (NLRs) ZAR1	Calcium channel	*Arabidopsis thaliana*	Calcium Influx	Plasma membrane	Bi et al., 2021
4.	1	Toll-like interleukin-1 receptor (TIR-NLRs)	Calcium channel	*Arabidopsis thaliana*	Calcium Influx	Plasma membrane	Ma et al., 2020
5.	1	TIR-NLR ROQ1 (recognition of XopQ 1)	Calcium channel	*Nicotiana benthamiana*	Calcium Influx	-	Martin et al., 2020
6.	3	Glutamate receptors like receptors (AtGLR1.4, AtGLR3.3, and AtGLR3.4)	Calcium channel	*Arabidopsis thaliana*	Calcium Influx	Root and shoot tissue (plasma membrane)	Qi et al., 2006; Tapken et al., 2013; Forde and Roberts, 2014
7.	1	Two-pore channels (TPCs)(OsTPC1)	Calcium channel	*Oryza sativa*	Calcium Influx	Plasma membrane	Kurusu et al., 2005
8.	1	AtTPC1	Calcium channel	*Arabidopsis thaliana*	Calcium Influx	Vacuolar membrane	Ranf et al., 2008
9.	–	Hyperpolarization-activated influx of Ca^2+^	Ca^2+^/Ba^+^ Exchanger	*Lycopersicon esculentum L.*	Calcium Influx	Plasma membrane	Gelli and Blumwald, 1997
10.	1	HYPERPOLARIZATION-ACTIVATED Ca^2+^CHANNELs (HACCs) CAX3	Ca^2+^/H^+^ Exchanger	*Arabidopsis thaliana*	Calcium Influx	Tonoplast	Manohar et al., 2011
11.	1	*Nicotiana benthamiana* Ca^2+^-ATPase1 (NbCA1)	Ca^2+^/ATP pump	*Nicotiana benthamiana*	Calcium Efflux	Plasma membrane	Zhu et al., 2010
12.	2	*Arabidopsis* Ca^2+^-ATPase (ACA4 and ACA11)	Ca^2+^/ATP pump	*Arabidopsis thaliana*	Calcium Efflux	Vacuolar membrane	Boursiac et al., 2010
13.	1	Soybean Ca^2+^-ATPase (SCA1)	Ca^2+^/ATP pump	*Glycine max*	Calcium Efflux	Plasma membrane	Chung et al., 2000

One of the most well-known calcium sensors in plants is the calmodulin (CaM) protein. CaM binds to calcium ions in a calcium-dependent manner, leading to a conformational change that allows it to interact with downstream effectors. CDPKs are a group of calcium-dependent protein kinases involved in several physiological processes, including stress response, development and hormone signaling (Cheng et al., 2002). CBL-interacting protein kinases (CIPKs) are another group of calcium sensors that interact with calcium-binding proteins called CBLs to regulate downstream signaling events (Kudla et al., 2010). The binding of CBLs to CIPKs is calcium-dependent, and the activation of CIPKs is induced by this interaction. Downstream targets, such as ion channels, transporters, transcription factors and enzymes, are phosphorylated by CIPKs as a consequence (Evans et al., 2016).

Factors like greenhouse conditions, extreme temperatures, drought stress, and chelation can hinder calcium uptake, leading to symptoms like black spots, bushy morphology, and stunted growth (Bose et al., 2011). Therefore, a critical study of the process of calcium transport, storage, and signaling is crucial for developing stress tolerant-high yielding crops. In this review, we aim to provide a comprehensive overview of calcium signaling molecules involved in different kind of plant-microbe interactions. We explore the emerging perspectives and discuss the future directions and challenges for harnessing calcium signatures to generate climate smart crops.

## Calcium signaling in plant responses to microbial pathogens

Calcium signaling is an essential factor of plant-microbe interactions, especially in response to microbial pathogens. When pathogens interact with host plants, they may trigger an influx of calcium ions (Ca^2+^) into host cells, which activates various defense responses. Calcium signaling pathways can also play a role in mediating communication and coordination between microbes, as quorum sensing systems can involve calcium signaling (DeFalco et al., 2023).

When plants encounter pathogens, an early signaling event is an increase in cytosolic Ca^2+^ concentration. The immune system of plant comprises two distinct pathways; Pathogen-Associated Molecular Patterns (PAMP)-Triggered Immunity (PTI) and Effector-Triggered Immunity (ETI). These pathways have different Ca^2+^ signatures. The PAMPs are recognized by Pattern Recognition Receptors (PRRs) triggering PTI, which induces a rapid and brief influx of Ca^2+^ from extracellular sources. This event activates downstream signaling pathways resulting in the expression of defense-related genes, ROS production and callose deposition. ETI is initiated when plants perceive specific pathogen effectors that disrupt host defenses. This recognition results in sustained elevations of Ca^2+^ and the activation of downstream signaling pathways, transcription factor activation, mitogen activated protein kinase cascade and hypersensitive response (Tian et al., 2020).

Plant-microbe interactions are initiated through the recognition of microbial-associated molecular patterns (MAMPs) or damage associated molecular patterns by plant pattern recognition receptors (PRRs) (Zipfel, 2014). Notably, the recognition of bacterial flagellin or flg22 minimal epitope involves the flagellin-sensitive 2 (FLS2) pattern recognition receptor, whereas LysM-receptor kinase 5 (LYK5) receptor complex and chitin elicitor receptor kinase 1 (CERK1) are crucial for sensing fungal chitin (Sun et al., 2013; Cao et al., 2014). Furthermore, the plant endogenous peptide 1 receptor (PEPR1) and PEPR2 function as Damage Associated DAMP receptors, perceiving host-derived DAMPs that are released during pathogen or insect attacks (Krol et al., 2010). Rhizobia and mycorrhizal fungi communicate their presence to host plant roots using specialized small molecules known as Nod factors and Myc factors, respectively, which are recognized by specific PRRs (Yuan et al., 2017).

**Figure 2:**
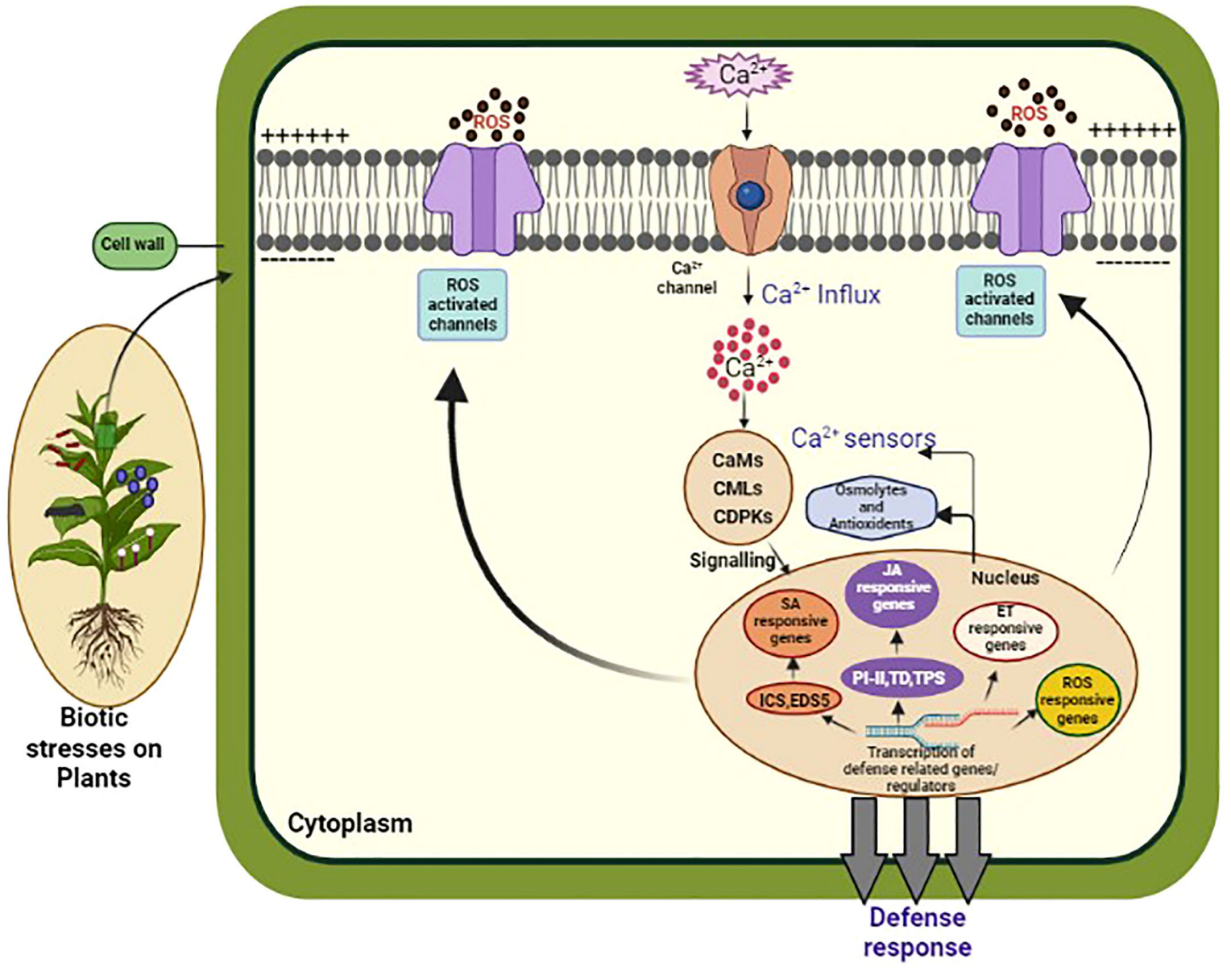
This diagram illustrates the Ca^2+^ signaling pathway in plants. Calcium ions (Ca^2+^) are important signaling molecules involved in various plant processes, including biotic and abiotic stress responses, growth, and development. In response to a stimulus, Ca^2+^ channels on the plasma membrane open, allowing Ca^2+^ to enter the cytosol. This leads to the activation of Ca^2+^ binding proteins, such as calmodulins (CaMs), and Ca^2+^ dependent protein kinases (CDPKs), which in turn activate downstream signaling pathways. These pathways can trigger gene expression changes, ion channel regulation, and activation of enzymes involved in secondary messenger production. The Ca^2+^ signaling pathway is tightly regulated by Ca^2+^ ATPases, which remove Ca^2+^ from the cytosol, and Ca^2+^ binding proteins, which buffer the Ca^2+^ concentration to prevent excessive Ca^2+^ accumulation and toxicity. Understanding the intricacies of Ca^2+^ signaling in plants is crucial for developing strategies to enhance plant growth, improve stress tolerance, and combat diseases.

Upon the detection of microbial-associated molecular patterns or damage associated molecular patterns by PRRs, there is a swift and temporary surge in cytosolic Ca^2+^ concentration. PRRs can directly activate calcium-permeable channels. For example, upon activation, FLS2 interacts with *Arabidopsis*-autoinhibted Ca^2+^ ATPase 8 (ACA8) and ATPase10, which acts as Ca^2+^ pumps situated in plasma membrane (Ma et al., 2013). Similarly, during fungal-plant interactions, CERK1 forms an association with Annexin1, which acts as a calcium-permeable channel (Wang et al., 2016). Notably, the presence of 8-mer chitin induces the accumulation of Annexin 1 (ANN1) protein, suggesting a potential supplementary role in chitin-triggered innate defense signaling. Additionally, PRRs- induces calcium influx may indirectly activate other signaling molecules, such as reactive oxygen species (ROS) and cyclic nucleotides, which in turn contribute to the overall defense response (Yuan et al., 2017) (**Table 2**; **Figure 2**). Moreover, calcium signaling pathways in plants can also be influenced by factors like abscisic acid (ABA), which induces stomatal closure during pathogen attacks and abiotic stress, thereby limiting pathogen entry and spread. Understanding the intricate role of calcium signaling in plant responses to microbial pathogens holds significant implications for developing strategies to enhance plant resistance and improve crop protection (Upadhyay, 2022).

**Table 2:** The involvement of various calcium signaling molecules in response to biotic stress, their role in stress response, and downstream effectors in plant-microbe interactions.

Calcium Signaling Molecule	Type of Stress	Role in Stress Response	Plant Response	Calcium Channels/Transporters	Calcium Signaling Pathway	Calcium Sensors	Downstream Effectors	References
Calmodulin (CaM)	Biotic stress	Activation of defense responses	Increased resistance to pathogens	GLR, CNGC	ROS, MAPK	CDPKs	Phytohormones, pathogenesis-related proteins	Ma et al., 2009; Reddy et al., 2011; Ma and Berkowitz, 2012
Calcium-dependent protein kinases (CDPKs)	Biotic stress	Activation of defense responses	Increased resistance to pathogens	CML, CDPK	Nuclear-localized Ca^2+^-CaM	CBL, CIPK	Phytohormones, transcription factors, pathogenesis-related proteins	Luan, 2009; Zou et al., 2010; Dubiella et al., 2013
Calcium and Calmodulin-dependent protein kinases (CIPKs)	Biotic stress	Activation of defense responses	Increased resistance to pathogens	CML, CIPK	Nuclear-localized Ca^2+^-CaM	CBL, CDPK	Phytohormones, transcription factors, pathogenesis-related proteins	Pandey et al., 2004; Xiang et al., 2007; Jiang et al., 2013
Calcium-dependent protein phosphatases (PP2Cs)	Biotic stress	Regulation of defense responses	Increased or decreased resistance to pathogens depending on the PP2C isoform	CNGC	Nuclear-localized Ca^2+^-CaM	CBL, CIPK	Phytohormones, transcription factors, pathogenesis-related proteins	Luan, 2009; Cutler et al., 2010
Annexins	Biotic stress	Regulation of defense responses	Increased or decreased resistance to pathogens depending on the Annexin isoform	CNGC	Nuclear-localized Ca^2+^-CaM	CBL, CIPK	Phytohormones, transcription factors, pathogenesis-related proteins	Batistic and Kudla, 2004; Mori et al., 2006; Liao et al., 2011
Calcium-binding EF-hand proteins	Biotic stress	Regulation of defense responses	Increased or decreased resistance to pathogens depending on the EF-hand protein isoform	CNGC	Nuclear-localized Ca^2+^-CaM	CBL, CIPK	Phytohormones, transcription factors, pathogenesis-related proteins	Laxalt and Munnik, 2002; Pandey et al., 2007
Calcineurin B-like proteins (CBLs)	Biotic stress	Regulation of stress responses	Increased or decreased resistance to pathogens depending on the CBL isoform	CNGC	Nuclear-localized Ca^2+^-CaM	CIPK, PP2Cs	Phytohormones, transcription factors, pathogenesis-related proteins	Zhang and Poo, 2001; Ma et al., 2009

## Calcium channels

Several channel proteins facilitate the exchange of calcium ions in plants, including cyclic nucleotide-gated channels (CNGCs), two pore channels 1 (TPC1), glutamate receptors (GLRs) and annexins. In *Arabidopsis*, for example, around 150 cation transporters exist, and 20 of these are CNGC-class calcium transporters that sense intracellular levels of cyclic nucleotide monophosphates (cNMPs), such as cAMP and cGMP, to regulate Ca^2+^ levels and transduce different signaling events (Xu et al., 2022).

Several CNGC channels in *Arabidopsis* have been identified to play significant roles in the immune response against pathogens. CNGC2 and CNGC4 were found to trigger ROS generation and ETI response upon recognition of flg22, a bacterial PAMP (Eichstadt et al., 2021). Additionally, CNGC2/defense, no death1 (DND1) regulates intracellular nitric oxide levels and defense responses, controlling HR-mediated cell death (Zhao et al., 2021). CNGC11 and CNGC12 participate in caspase-dependent programmed cell death (PCD) in response to pathogenesis, while CNGC20 has a significant role in plant immune response by interacting with CNGC19 BOTRYTIS INDUCED KINASE 1 (BIK1) (Ren et al., 2021). Interestingly, the cpr22 mutant contains a chimeric CNGC channel (AtCNGC11/12) resulting from a deletion between AtCNGC11 and AtCNGC12, which leads to constitutive expression of PR genes22, and thus provides resistance to virulent *Hyaloperonospora parasitica.* The cpr22 mutant is semi-dominant, and homozygosity for the mutant allele is lethal (Clough et al., 2000; Moeder et al., 2011). Moreover, the Ca^2+^ dependent cell-death observed in the cpr22 mutant suggests the involvement of calcium ions in this process (Urquhart et al., 2007).

Plant GLR-type Ca^2+^ receptors are a common feature in plants and are structurally similar to ionotropic glutamate receptors found in animals. *Arabidopsis* contains 20 GLR-type Ca^2+^ transporters, with most of them performing developmental functions. While most studies on glutamate receptor-like proteins have focused on their role in ion homeostasis and neurotransmission in animals, recent research has highlighted their potential involvement in immune signaling and plant stress adaptations (Ghosh et al., 2022). Responsiveness to various abiotic stresses like cold, drought and salt, as well as biotic stress like infection with the fungal pathogen *Phytophthora sojae* has been demonstrated by GLRs in soybean (Zhang et al., 2021). An immune response has been observed in *Arabidopsis*, upon topical application of glutamate, as evidenced by the activation of various PTI-responsive gees, such as BAK1, BKK1. BIK1, PBL1 and CERK1, as well as chitin receptor LYSIN-MOTIF RECEPTOR-LIKE KINASE 5 (LYK5) and genes involved in the biosynthesis of plant hormone salicylic acid (SID2) (Bhar et al., 2023). The results suggest that GLRs have a previously unappreciated role in plant immune signaling and could open new avenues for research into the molecular mechanisms underlying plant stress responses.

Annexins are a conserved family of cytoplasmic proteins that interact with phospholipids in the cell membrane in a Ca^2+^-dependent or -independent manner. They play essential roles in regulating various cellular processes in plants, including responses to abiotic and biotic stresses as well as developmental processes (Gerke and Moss, 2002). The unique feature of annexins is their annexin core domain, a Ca^2+^ and membrane-binding module, which allows Ca^2+^ bound annexins to associate with membranes containing negatively charged phospholipids. This interaction between annexins and phospholipids is critical for their function in cellular processes.

In addition to the annexin core domain, each annexin contains a highly variable region known as the N-terminal interaction domain. This region serves as a binding site for cytoplasmic protein ligands that can be directed to membranes through the annexin-core-mediated phospholipid interaction. Due to their ability to interact with membranes and modulate calcium-dependent responses, annexins are versatile regulators involved in a wide range of cellular activities, contributing to the overall functioning and adaptation of plants in their environment. Mechanosensitive channels are a class of ion channels found in various organisms, including plants, animals, bacteria and fungi (Martinac, 1993). These channels are known for their ability to respond to mechanical forces and changes in membrane tension. One significant aspect of their function is their role in mediating calcium signaling in response to mechanical stimuli. When a cell experiences mechanical stress or changes in membrane tension, mechanosensitive channels are activated, allowing the influx of ions, including calcium (Ca^2+^), into the cell. This increase in intracellular calcium concentration serves as a crucial second messenger, initiating a cascade of signaling events that regulate various cellular processes. In plants, mechanosensitive channels have been extensively studied, particularly in *Arabidopsis* (Hamilton et al., 2015). They include mechanosensitive-like channels (MSLs), Mid1-complementing activity channels (MCAs), and two-pore potassium (TPK) families. The mechanosensitive-like channels (MSLs) located on mitochondria, chloroplasts, and the plasma membrane (PM) possess transmembrane domains that form a pore, allowing the influx of calcium ions upon mechanical stimuli. The MSLs lacking voltage sensor regions respond solely to mechanical cues, highlighting their specialized role in mechanotransduction. Mid1-complementing activity channels (MCAs) are formed by subunits containing a single transmembrane domain. These subunits come together to form a pore at the cell membrane. When activated by mechanical forces, MCAs facilitate calcium entry, contributing to calcium signaling (Shigematsu et al., 2014). The two-pore potassium (TPK) channels, as the name suggests, contain two K^+^ channel pore domains. They exhibit unique structural features and are involved in both potassium and calcium transport. TPKs can respond to mechanical stress and osmotic changes, modulating calcium signaling and ion fluxes in response to these stimuli. The activation of mechanosensitive channels and subsequent calcium signaling play vital roles in diverse physiological processes. For instance, in plants, they are involved in touch and gravity sensing, osmoregulation, and responses to environmental cues such as wind or touch (Becker et al., 2004).

OSCAs are a family of conserved channels found in eukaryotes. These channels have nine transmembranous domains, and a pore domain is located between the eighth and ninth domains. In Ar*abidopsis*, there are 15 OSCA genes encoded in the genome. Two of these genes, OSCA1.1 and OSCA1.2, have been identified as being involved in the transport of calcium ions (Yuan et al., 2014). On the other hand, Piezo channels are composed of 2000 to 4000 amino acid residues and are predicted to have 20 to 40 transmembranous domains (Hamilton et al., 2015). Both OSCAs and Piezo channels are mechanosensitive, meaning they respond to mechanical forces.

## From calcium sensors to signaling pathways: deciphering the intricate language of calcium signatures

Conformational changes induced by calcium play a crucial role in relying of Ca^2+^ signals to downstream signaling components, such as CBPs, in response to biotic and abiotic stress involving different kind of calcium receptors. On the other hand, CBLs relay Ca^2+^ signals to CIPKs, which regulate various physiological processes in plants. While CaM/CMLs and CBLs are crucial for Ca^2+^ signaling, within the same molecule, CPKs contain both a kinase domain and a CaM like calcium sensor domain. Therefore, they can directly decode Ca^2+^ signaling into phosphorylation events, providing a more efficient and rapid response to Ca^2+^ signals (Seybold et al., 2014; Verma et al., 2022).

## Calmodulin (CaM) and CaM-like proteins (CML)

Plants have various forms of Calmodulins, also termed CaM-like proteins (CMLs), contain EF-hand motifs, which are common calcium-binding structural domains found in calcium sensor proteins. In CMLs, the EF-hand motifs are typically present in pairs, and each motif consists of approximately 30 amino acids, forming a helix-loop-helix structure. The functional significance of these EF-hand motifs in CMLs lies in their calcium-binding properties and their role as calcium sensors in plant cells (Mohanta et al., 2015; Kaur and Upadhyay, 2022). The Calmodulin and CaM-like protein family members may have functional redundancy, individual CaM/CML gene expression deregulation or loss of CML function in mutated plants which can impact pathogenic defense responses. The Calmodulin and CaM-like protein family members play a crucial role in mitigating different abiotic stresses too (Raina et al., 2021b). Gain-and loss-of-function genetic approaches have further strengthened the role of CMLs in plant immunity. Several studies have explored the calcium sensors like CaMs and CMLs for their role in plant defense responses. The overexpression of soybean CMLs in tobacco plants has shown improved resistance against various pathogens, including bacteria, viruses, and fungi. The overexpression of CMLs in Transgenic tobacco plants with increased expression of GmCaM4 or GmCaM5 demonstrate spontaneous necrotic lesions and constitutive expression of systemic acquired resistance (SAR)-associated genes, which occurs independently of salicylic acid (SA) production (Heo et al., 1999). Additionally, these genetically modified plants display heightened resistance against a diverse range of pathogens, including *Phytophthora parasitica* var. *nicotianae, Pseudomonas syringa*e pv. *tabaci*, and Tobacco mosaic virus. These findings indicate that specific CaM isoforms are involved in an SA-independent signaling pathway that triggers disease resistance in the transgenic tobacco plants.

Plants possess a diverse range of CaM-like proteins (CMLs) that are distinct from typical CaMs. In *Arabidopsis thaliana* alone, there are 50 members of the CML family, each of which may have a specific role in plant physiology due to their varying expression patterns in response to developmental stages, tissues, and environmental stimuli. Recent research has highlighted the critical role of CMLs in plant immunity, where they act as essential Ca^2+^ sensors involved in defense mechanisms against microbial pathogens and herbivores. Numerous studies have demonstrated the importance of individual CaM/CML gene expression in plant defense responses to various pathogens. Gain-of-function experiments have demonstrated that overexpression of specific CMLs, such as soybean CMLs, can boost plant tolerance to a broad range of pathogens. For example, overexpressing soybean CMLs in tobacco results in increased resistance to various pathogens, while overexpression of a typical CaM does not have the same effect, suggesting that CMLs are selectively activated in response to pathogen invasion.

The reduction of hypersensitive response was demonstrated in tomato when APR134 was silenced (Chiasson et al., 2005). Conversely, the stimulation of HR was observed to an avirulent strain of *P. syringae* by overexpressing the APR134 ortholog from *Arabidopsis* CML43. Studies have shown crucial roles in regulating plant defense against various pathogens in *Arabidopsis* are played by calcium binding proteins such as CML8, CML9, CML24 and CML41. The hypersensitive response is impaired and nitric oxide production is reduced upon recognition pf pathogen-associated molecular patterns when CML24 is knocked out. Plant defense against different strains of *Pseudomonas syringae* is positively regulated by CML9 and CML8 and CML8 functions mainly through salicylic acid (SA)-dependent pathways. Furthermore, defense against *P. syringae* is positively regulated by CML41 through the facilitation of plasmodesmata closure in response to bacterial flagellin (Leba et al., 2012; Zhu et al., 2017). Resistance to *Spodoptera littoralis* is enhanced by CML42, whereas defense against the same insect herbivory is positively influenced by CML37, according to Scholz et al. (2014). These calcium-binding proteins act as calcium sensors, enabling changes in calcium levels to be perceived by plants and translated into various signaling pathways that activate defense responses against pathogens. Overexpression of SCaM-5 in *Arabidopsis* also enhances resistance to *Pseudomonas syringae* infection, while overexpression of a typical CaM (SCAM-1) does not (Bouche et al., 2002; Park et al., 2004). SCaM-4 is a calcium-binding protein, and its overexpression triggers molecular and cellular responses that strengthen the plant’s defense mechanisms. This enhanced resistance could potentially offer a valuable strategy for improving soybean crop protection against these harmful fungal diseases (Aldon et al., 2018). Additionally, overexpressing SCaM-4 in soybean stimulates resistance to fungal pathogens *Alternaria tenuissima, Phomopsis longicolla and Phytophthora sojae* (Rao et al., 2014).

Loss-of-function genetic studies have provided valuable insights into the roles of different CMLs in plant immunity. The reduction in expression of pathogen-induced CaM isoforms, specifically NtCaM1 and NtCaM13, had distinct effects on disease resistance in tobacco (Takabatake et al., 2007). Silencing NtCaM13 increased the plant’s vulnerability to viral, bacterial, and fungal pathogens, while knockdown of NtCaM1 did not. Conversely, when pepper CaM1 was transiently overexpressed, it stimulated the production of reactive oxygen species and NO, resulting in the appearance of HR-like lesions and the activation of defense-related genes in pepper leaves. This led to local resistance against bacterial pathogens. Zhu et al. (2017) demonstrated that silencing of APR134 in tomato suppresses the hypersensitive response (HR) in tomato plants. They also observed that the overexpression of *Arabidopsis* CML43 ortholog of APR134 stimulates the HR in response to an avirulent strain of *P. syringae.* Similarly, knockout of CML24 in *Arabidopsis* reduces nitric oxide production and impairs the HR response after PAMP recognition (Dubreuil-Maurizi et al., 2011). CML8 and CML9 act as positive regulators of plant defense against different strains of *P. syringae*, with CML9 also contributing to plant immune responses despite its initial identification as a gene involved in plant responses to abiotic stress (Delk et al., 2005; Luan et al., 2009; Reddy et al., 2011). Recent research by Boudsocq and Sheen (2013) has identified plasmodesmal-localized CML41 as a positive regulator of defense against *P. syringae*. The Ca^2+^ signaling is also involved in plant responses to herbivores. Studies have shown that Arabidopsis CML42 knockout harbor increased resistance against *Spodoptera littoralis*, with an upregulation of JA-responsive genes and accumulation of glucosinolates. Furthermore, Zhang et al. (2022a) reported that silencing SlCML55 in tomatoes (*Solanum lycopersicum*) leads to higher tolerance against the oomycete pathogen *Phytophthora capsici*, as this CML was found to exert negative control on the activation of PR genes.

## Calcium-dependent protein kinases (CDPKs)

CDPKs play a crucial role in plant defense signaling against various biotic and abiotic stresses. Unlike other Ca^2+^ decoders, CDPKs integrate both Ca^2+^ sensing and downstream signal propagation capabilities into a single module. In angiosperms, the number of CDPK members is around 30, and they are organized into four distinct groups based on their family architecture. CDPKs or calcium dependent protein kinase are diverse group of serine/threonine protein kinase found in plants. They play crucial roles in regulating various aspects of plant growth development and responses to abiotic and biotic stresses. CDPKs consists of three main structural components: a variable N-terminal, a central kinase domain, and an activation domain. It typically consists of an auto-inhibitory pseudo-substrate linked to a CaM like domain containing four EF- hands and acts as calcium sensor. When calcium ions bind to the EF-hands, a conformation change occurs, relieving the auto-inhibition and enabling kinase activation. This allows the kinase to become active and initiate downstream phosphorylation events, which can lead to various cellular responses such as gene expression, ion fluxes, and protein degradation (Hu et al., 2016; Kaur and Upadhyay, 2022). The role of calcium-dependent protein kinases (CDPKs) in potatoes and their involvement in various biological processes, including hormone signaling, plant growth, and responses to abiotic and biotic stresses have been demonstrated. Fantino et al. (2017) studied StCDPK7 and found high transcript levels in swollen stolons, roots, and mini tubers, with induced expression upon *Phytophthora infestans* infection in systemic leaves. Surprisingly, StCDPK7 displayed cytosolic/nuclear localization despite a predicted chloroplast transit peptide. The recombinant protein, StCDPK7:6xHis, exhibited Ca^2+^-dependent kinase activity and could phosphorylate phenylalanine ammonia lyase, an enzyme involved in plant defense response. Another study Mori et al. (2006) provides compelling evidence for the involvement of CDPK5 (also known as CPK6) in tomato (*Solanum lycopersicum*) in regulating guard cell S-type anion- and Ca^2+^-permeable channels, which are crucial for stomatal closure. The research demonstrates that CDPK5/CPK6 plays a pivotal role in ABA-mediated signaling pathways, leading to the activation of downstream defense responses in guard cells. This example sheds light on the specific functions of CDPKs in tomato and highlights their significant contribution to enhancing plant defense against pathogens.

The plant immune system is impacted by the presence of CDPKs, as defense gene expression upon bacterial flagellin perception is positively regulated by them. PTI-induced resistance against *Pseudomonas syringae* is collectively contributed by CDPK4, CDPK5, CDPK6 and CDPK11, while AtRBOHD, the primary reactive oxygen species producing enzymes involved in immunity, can be phosphorylated by CDPK5, CDPK6, CDPK11 and CDPK4 (Boudsocq et al., 2010). The phosphorylation of the HsfB2a transcription factor regulates the induction of the PDF1.2 defense gene after caterpillar *Sodoptera littoralis*-induced wounding, and this is facilitated by CDPK3 and CDPK13 (Kanchiswamy et al., 2010). Disease resistance against *Magnaporthe oryzae* in rice is enhanced through the overexpression of full-lenth OsCDPK4, resulting in elevated basal levels of salicylic acid and augmented defense gene induction (Bundo and Coca, 2016).

Plant cell death in response to pathogens is controlled by CDPKs as well. In *Nicotiana* sp., the programmed cell death response triggered by the perception of the *Cladosporium fulvum* race-specific Avr4 or Avr9 elicitors requires CDPK2 and CDPK3. In *Arabidopsis, the* onset of hypersensitive response upon challenge with avirulent *P. syringae* and subsequent NLR-mediated effector recognition is controlled by CDPK1 and CDPK2 (Romeis et al., 2001). Several constitutively active CDPKs harbor cell death-inducing activity. Cell death-inducing activity is harbored by several constitutively active CDPKs. The activation domains of auto-active CDPKs are absent, and their active state does not rely on calcium ion inputs. Cell death and kinase activity are both required when CDPK5 is expressed in *Arabidopsis* leaf protoplasts. The cell death-inducing activity of barley *CDPK4* or *Arabidopsis CDPK5*, when transiently expressed, has been observed in tobacco leaves. It appears that the activation of a specific CDPKs alone is not sufficient to trigger PCD, as not all auto-active configurations of tested CDPKs induce PCD (Dubiella et al., 2013).

## Calcineurin B-like proteins (CBLs) and CBL-interacting protein kinases (CIPKs)

Calcineurin B-like proteins (CBLs) and CBL-interacting protein kinases (CIPKs) have emerged as a new class of plant calcium sensors that play a crucial role in response to both biotic and abiotic stresses (Thakur and Negi, 2021). *Arabidopsis* has 10 CBLs and 25 CIPKs, while maize has 12 CBL genes that have been linked to abiotic stress tolerance. However, recent genome-wide analysis of *Lagerstroemia indica* (crape myrtle) revealed 37 CIPKs, indicating their abundance in plants (Yu et al., 2022).

The role of CBL-CIPKs in biotic stress response is currently receiving increased attention, although their functions in abiotic stress tolerance are well established (Ma et al., 2020; Plasencia et al., 2021; Xiaolin et al., 2022). In rice, the upregulation of OsCIPK14 and OsCIPK15 were observed in response to PAMP treatment, resulting in resistance through the activation of ROS-mediated HR and cell dealth (Kurusu et al., 2010). Conversely, a recent study in wheat demonstrated that CIPK14 negatively regulates resistance against rust fungi, specifically *Puccinia striiformis* f. sp. tritici (*Pst*) (He et al., 2023).

In addition to biotic stress response, CIPKs have also been linked to nitrogen uptake and root development. Recent research has revealed the involvement of CmCIPK23, a CIPK from Chrysanthemum, in the regulation of CmTGA1 and activation of nitrogen uptake during root development (Liu et al., 2022). Interestingly, TGA transcription factors, which are essential for NPR1-dependent PR1 activation, may also be linked to pathogenesis, suggesting a potential role for CBLs and CIPKs in plant defense mechanisms that require further exploration. Studies have identified the role of CBLs and CIPKs in stress responses in other plant species as well. In pepper (*Capsicum annum* L.), nine CaCBLs and twenty-six CaCIPKs were induced under stress conditions. CIPK1 specifically participates in biotic stress responses in *C. annum*, as shown by the increased sensitivity of cacipk1 mutant lines to the fungal pathogen *Phytophthora capsici*. Conversely, CaCIPK1-OE lines exhibit heightened defense activity, accompanied by H_2_O_2_ accumulation and cell death (Ma et al., 2019). In wheat (*Triticum aestivum*), TaCBL4 has been shown to be involved in defense responses against *Puccinia striiformis* f. sp. tritici infection. The upregulation of TaCBL4 following infection and increased susceptibility in the loss-of-TaCBL4 function mutants suggest its role in the defense mechanism. TaCBL4 interacts with TaCIPK5, indicating their joint participation in positive defense mechanisms (Liu et al., 2018). The enhanced tolerance to *Pst in* wheat can be achieved by overexpressing TaCIPK10, which triggers hypersensitive cell death and accumulation of reactive oxygen species (ROS). TaNH2, a counterpart of *Arabidopsis* NPR3/4, has been shown to play a crucial role in salicylic acid (SA) signaling and collaborate with TaCIPK10 in the defense response by interacting and phosphorylating it (Liu et al., 2019). In tomato, SlCBL10 interacts with SlCIPK6 to regulate its kinase activity. Overexpressing SlCIPK6 in *Nicotiana benthamiana* induces ROS production by regulating NbRBOH and the kinase activity of SlCIPK6. The complex of SlCBL10-SlCIPK6 interacts with the downstream protein RBOH, which leads to an increase in ROS, thereby contributing to plant immunity (de la Torre et al., 2013). These findings emphasize the essential role of Ca^2+^ signaling components in stress responses and suggest their potential application in the development of stress-tolerant crops.

## Modulation of calcium signaling by microbial effectors

The Ca^2+^ ion concentration inside the cell system changes, as soon a microbe is sensed in the external milieu. An elevation in Ca^2+^ ions has been observed during symbiotic or pathogenic interaction of microbes with plants. The Ca^2+^ influx and the oxidative burst are the plant’s first and foremost immunologic response to pathogenic elicitors. Increased Ca^2+^ ions have been known to be associated with the gene expression for suitable defense mechanism (Gilroy et al., 1990). The involvement of calcium signaling during plant-microbe interactions to bring about pathogenic or symbiotic outcome is compiled in **Table 3**. The effectors are protein molecules of microbial origin, which upon suitable molecular interactions, bring about either a pathogenic or a defense response. These microbial effectors are responsible for debilitating the host plant to bring about an effective pathogenesis. These effectors also aid in host invasion and nutrient acquisition for the pathogen.

**Table 3:** The involvement of calcium signaling in plant-microbe interactions.

Study	Organism	Interaction	Key findings
Tang et al. (2022)	*Oryza sativa*	*Xanthomonas oryzae pv. Oryzicola* (Xoc) bacterial interaction	Calcium signaling pathways involved in the plant defense against bacterial pathogens *Xanthomonas orzyae* pv, *oryzicola* (Xoc).
Zhang et al. (2022b)	*Medicago sativa* L.	*Fusarium proliferarum L1* fungal interaction	CDPKs, CIPKs involved in the regulation of plant defense against *Fusarium proliferarum.*
Wang et al. (2022)	Apple (*Malus* sp.)	*Valsa mali* fungal interaction	CNGCs and CDPKs are involved with the plant’s defense response to *Valsa mali* infection, providing valuable insights into the mechanisms underlying apple’s resistance against Valsa canker.
Sun et al. (2022a)	*Arabidopsis thaliana*	*PAMPs* bacterial interaction	CaM binding proteins 60- LIKE G (CBP60g), CaM, CIPK5, CDPK4, 5, 6, and 11 involved in plant-microbe interactions and enhanced plant immune responses against microbial pathogens.
Sun et al. (2022b)	*Poa pratensis* L.	*Blumeria graminis* (DC.) Speer fungal interaction	CDPKs involved in the host-pathogen interaction and contributes to the development of strategies for powdery mildew management in Kentucky bluegrass.
Yuan et al. (2022)	*Arabidopsis thaliana*	Pathogen interaction	CaM and Ca^2+^ pathways involved in the plant’s immune response and provided insights into the complex network of plant defense against different pathogens.
Bai et al. (2022)	*Arabidopsis thaliana*	*Pseudomonas syringae* and *Botrytis cinerea*	Calcium signaling pathways involved in the plant defense against bacterial pathogens Pseudomonas syringae.
Li et al. (2022)	*Citrus*	*Penicillum digitatum* and *P. italicum*	CaMKs involved in the plant’ defense mechanism against fungal pathogens *Penicillum digitatum* and *P. italicum.*
Zhu et al. (2021)	*Leifsonia xyli* subsp. *Xyli *(Lxx)	*Saccharum offinarum* L. bacterial interaction	Calcium-dependent protein kinase involved in the regulation of plant defense responses against sugarcane ratoon stunting disease.
Mishra et al. (2022)	*Lens culinaris*	*Rhizoctonia bataticola *root interaction	CDPKs, CaMKs involved in the regulation of plant defense responses against *Sporisorium destreuns*.
Jin et al. (2021)	*Panicum miliaceum* L.	*Sporisorium destreuns* fungal interaction	CDPKs & calcium signaling involved in the regulation of smut disease in *Panicum miliaceum* L and its potential impact of crop yield.
Meng et al. (2021)	*Nicotiana tobacum*	*Phytopthora nicotianae* fungal interaction	CNGCs, CDPKs involved in the pathogen’s virulence and the plants defense response, contributing to a better understanding of the molecular mechanism between underlying the interaction between tobacco and *Phytopthora* nicotianae.
Shu et al. (2020)	*Citrus sinensis*	Arbuscular mycorrhizal fungi (AMF)	Calcium signaling plays a crucial role in the response to AMF and drought stress.
Wang et al. (2019)	*Arabidopsis thaliana*	*Sclerotinia sclerotiorum* (Lib.)	Calcium signaling plays an important role in regulation of plant defense against *Sclerotinia sclerotiorum.*
Gonçalves et al. (2019)	*Vitis vinifera*	Interaction between *Vitis vinifera *(grapewine) and the fungal pathogen*Lasiodiplodia theobromae*	By using dual RNA- Seq approach, study aimed to understand the molecular basis between the plant and pathogen and identified genes and calcium signaling pathways involved in the grapewine response to *Lasiodiplodia theobromae* and provide insignificant pathogenicity mechanism of the fungus.
Kamal et al. (2019)	*Gossypium hirsutum*	Begomoviruses	The study aimed to understand the molecular basis of virus-host interactions and the role of CLCuMB in disease development and identified host factors involved in viral replication and movement, shedding light on the mechanisms of virus infection and disease progression in cotton plants.
Lu et al. (2019)	*Triticum aestivum *L.	*Triticum aestivum* and fungal pathogen*Rhizoctonia cerealis*	Identified TaCML36 gene associated with *Triticum aestivum* resistant to fungus *Rhizoctonoia cere*lis pathogen and provide insides into the molecular mechanism underlying the plant pathogen interaction.
Jiang et al. (2019)	*Arabidopsis thaliana*	Fungi-root interaction	Calcium signaling is involved in the regulation of plant defense responses against the fungal pathogen *Fusarium oxysporum.*
Wan et al. (2018)	Tomato	Bacteria-leaf interaction	Calcium signaling is involved in the regulation of defense responses against *Pseudomonas syringae* infection in tomato plants.
Yu et al. (2018)	*Medicago truncatula*	Rhizobium	Calcium-dependent protein kinase (CDPK) involved in the plant defense against rhizobium in *Medicago truncatula*.
Xiong et al. (2018)	Strawberry	*Botrytis cinerea*	RNA- Seq analysis revealed that infection of mature strawberry fruit by pathogen led to significant induction of calcium signaling pathway genes, highlighted their specific *B. cinerea* strawberry interaction.
Chen et al. (2017)	*Arabidopsis thaliana*	Fungi-root interaction	Calcium signaling is involved in the regulation of plant growth and defense responses during colonization by arbuscular mycorrhizal fungi.
Martínez-Medina et al. (2017)	*Medicago truncatula*	Fungi-root interaction	Calcium signaling is involved in the regulation of plant defense responses during colonization by arbuscular mycorrhizal fungi.
Kiep et al. (2015)	*Arabidopsis thaliana*	Bacteria-leaf interaction	Calcium signaling plays an important role in the regulation of plant defense responses against *Pseudomonas syringae* infection.
Bohm et al. (2014)	*Arabidopsis thaliana*	Bacteria-root interaction	Calcium signaling plays a crucial role in the early responses to bacterial colonization of plant roots.
Evangelisti et al. (2014)	*Arabidopsis* and *Nicotiana benthamiana* etc.	Plant-microbe or plant symbiotic interaction	Calcium signaling pathways involved in the plant defense against plant-microbe or symbiotic interaction.

The bacterial cells have been known to influence their positive or negative interaction by virtue of Ca^2+^ modulation. Grant et al. (2000) measured the cytosolic Ca^2+^ concentration in leaves of *Arabidopsis* plants, infected with *Pseudomonas syringae* using aequorin-mediated bioluminescence. The Ca^2+^ signal peaked after ~10 min and a second, Ca^2+^ signal was observed after 1.5–2 h, which was much stronger and robust. They studied avrRpm/RPM1 gene to gene interaction during early stages of hypersensitive response (HR) and observed a constitutively enhanced Ca^2+^ in cytosol. Since the Ca^2+^ signals are very localized and are influenced by other cellular and sub-cellular factors, they pioneered to use a whole plant system to explore calcium connections during plant-pathogen interaction. *Botrytis*–induced kinase 1 (BIK1) is transcribed in plants upon infection by *Botrytis* and defends plants from biotrophic and necrotrophic pathogens by regulating salicylic acid (SA) synthesis in plant cell (Veronese et al., 2006). It plays a role in regulation of Ca^2+^ influxes, triggered by membrane protein Flagellin 22 (flg22). It is also known to increase ROS generation by modulating the calcium concentration owing to site specific phosphorylation of NADPH oxidase RbohD (Li et al., 2014). Thor and Peiter (2014) used fluorescent genetically encoded Ca^2+^ indicators (GECIs) to investigate the oscillatory nature of flg22‐induced cytosolic Ca^2+^ signals at single cell level in the guard cells of the leaves of *Arabidopsis thaliana*. They observed that the flg22 could elicit oscillations in single cell and accordingly, all the oscillations occurring in all the cells contribute to the total oscillation of the whole plant. The pathogenic and mutualistic fungi too establish their relation with plants by altering Ca^2+^ efflux or influx. The cytoplasmic free calcium ([Ca^2+^]_cyt_) transiently increased to about 1 μM in transgenic Parsley expressing apoaequorin, upon treatment with Pep-13, an oligopeptide elicitor obtained from *Phytophthora sojae.* This peaked concentration of ([Ca^2+^]_cyt_), later returned to normal level of about 300 nM. This biphasic variation in calcium concentration was observed to be associated with oxidative burst and phytoalexin production (Blume et al., 2000). The transgenic cell suspension of *Nicotiana plumbaginifolia* expressing apoaequorin proteins have been studied for changes in ([Ca^2+^]_cyt_), when treated with cryptogein and oligogalacturonides, the elicitors of defense mechanism in plants. The elicitor treated cells showed Ca^2+^ influx and released calcium from internal stores (Lecourieux et al., 2002). The endophyte, *Piriformospora indica* is known to mitigate biotic and abiotic stresses, when co-cultured with plant roots. The subcellular levels of Ca^2+^ increased in roots of germinating seedlings of *Arabidopsis*, when treated with cell wall extract of *P. indica*, thereby establishing calcium connection between successful mutualism and stress mitigation (Vadassery et al., 2009). The PRRs and downstream signaling components such as receptor-like cytoplasmic kinases (RLCKs) are required for PTI (Ranf et al., 2014). The Ca^2+^-dependent protein kinase CPK28 degrades the excess BIK1 protein to maintain its optimal concentration. The *cpk28* knockout mutants accumulate an increased level of BIK1 protein while CPK28 over-expressing *Arabidopsis* plants have below optimal levels of BIK1 protein and are immune compromised. Therefore, Monaghan et al. (2015) concluded that CPK28 is a negative regulator of BIK1 and has a role in PAMP-induced Ca^2+^ burst.

## The role of endophytic microbes in calcium signaling

The endophytic microbes, usually fungal or bacterial, inhabit the internal tissues of plants but without causing disease. The endophytes reside in the seeds and along with the process of germination, they promote healthy growth of the plant. The endophytes are present in nearly all the plants. In some cases, they may have originated from soil, which upon successful interaction in the rhizosphere, colonize the plants internally to the benefit of the host. Likewise, the endophytes become an integral component of plants. These endophytes are beneficial to plants in multiparous ways like they enhance nutrients uptake by plant roots; strengthen the defense mechanism of plants; alleviate stress induced damages; modulate plant development and keep a check on the weed growth. Wais et al. (2000) injected oregon-dextran dye in the cells of root hairs by iontophoresis to demonstrate Ca^2+^ spiking in plant mutants lacking early symbiotic responses to bacteria. All mutant lines failed to form nodules. Five complementation groups were studied, and two mutants dmi1 and dmi2 were almost blocked in calcium spiking. Therefore, they concluded that *DMI1* and *DMI2* were involved upstream of the Ca^2+^ spikes during rhizobia symbiosis. Two years later, Wais et al. (2002) studied spatiotemporal Ca^2+^ spiking for analysis of the regulation of *nod* gene expression during *S. meliloti*-*M. truncatula* interaction. They differentiated the strains on the basis of their ability to produce Nod factor, which is attributed to the Ca^2+^ oscillations induced by them. Thus, two strains of *S. meliloti* (Rm1021 and Rm, 2011) had different kinetics of Ca^2+^ spiking. The strain Rm1021 elicited a robust Ca^2+^ spiking after an initial lag phase of 10–15 min, while no Ca^2+^ spiking was detected in the strain Rm2011. They also observed in their study that the calcium spiking triggered by bacteria is no different than the calcium spiking observed in response to purified nodulation factors. Therefore, it was concluded that the calcium spiking is a *nod* gene-dependent host response. The availability of genetically encoded Ca^2+^ probes facilitated the study of Ca^2+^ concentrations at the cell and organ level during symbiotic interaction of plants with microbes. Kosuta et al. (2008) compared and discriminated two types of symbiosis by virtue of Ca^2+^ oscillations induced by them. The two symbiotic pathways required both DMI1 and DMI2 for functional symbiosis and for Ca^2+^ oscillations. The results also indicate that Ca^2+^ oscillations are an essential and necessary process for rhizobial or mycorrhizal colonization of plant roots. It is notable that Ca^2+^ oscillations induced by symbiotic fungus differ in its amplitude and periodicity from the Ca^2+^ oscillations induced by Nod-factors. Sieberer et al. (2009) carried their research on *Medicago* plants to evaluate Ca^2+^ responses in nucleus of mutant lines defective for *NFP*, *DMI1*, and *DMI2.* These three genes function upstream of Ca^2+^ spiking in Nodulation factor (NF) mediated signal transduction. The defective mutant of *DMI3*, which encodes for CCaMK was also studied. When treated with NF elicitors, no change in Ca^2+^ concentration in nucleus of *nfp*, *dmi1*, and *dmi2* mutants was observed. However, the *dmi3* mutant showed an immediate surge in nuclear calcium spiking. Using a nucleoplasmin-tagged cameleon (NupYC2.1) coupled with mathematical modeling and time lapse imaging, they concluded that the initial spiking in Ca^2+^ originates in the vicinity of both sides of the nuclear envelope.

## Calcium signaling in beneficial plant-microbe interactions

The beneficial symbiotic and mutualistic associations of plants and microbes are also governed by Ca^2+^ signals. In rhizobia-legume symbiosis, Ca^2+^ signals are necessary for recognition and infection process for root nodule formation (Oldroyd, 2013). The infection process starts with the secretion of bacterial nodulation factors (NFs), which are recognized by plant receptors, resulting in a transitionary surge in cellular Ca^2+^ in plant cells (Gage, 2004). The Ca^2+^ signal transcriptionally activates the genes involved in the formation and differentiation of the nodules (Mitra et al., 2004).

Garcia et al., 2016 concluded that Ca^2+^ signaling is required for successful establishment of arbuscular mycorrhizal (AM) symbiosis as they investigated the mutualistic associations of plant roots and fungi of the *Glomeromycota phylum*. The hyphae enter the root cells and forms arbuscules, wherein nutrient exchange between the plant and the fungus takes place. The Ca^2+^ signals regulate this nutrient exchange and is involved in the establishment of the plant-fungus interface.

In mycorrhizal symbiosis, calcium signaling is essential for the recognition and coordination of the mutualistic association between plant roots and mycorrhizal fungi to establish a successful association. Flavonoid compounds released from plant roots signal compatible fungal species, while chitin oligosaccharides secreted by the fungal hyphae stimulate calcium signaling in plant cells. Calcium signaling activates downstream signaling pathways that lead to the formation of a symbiotic interface, where the exchange of nutrients between the plant and the fungus can occur. The nuclei in plants respond to rhizobacteria and AM fungi through the induction of a Ca^2+^ spiking response. This spiking in Ca^2+^ are recognized by the plant-specific CCaMK that contains a kinase domain and three Ca^2+^-binding EF hands. The activation of CCaMK is determined by free Ca^2+^ or activated calmodulin, while variations in the Ca^2+^ signatures determine whether the Ca^2+^ information is decoded into symbiosis or nodule formation. The autophosphorylation site of CCaMK is responsible for nodule formation, indicating that it acts as a regulatory switch.

During the establishment of a symbiotic relationship between plants and mycorrhizal fungi, transient changes in cytosolic calcium (Ca^2+^) occur, which indicate that host cells perceive the signaling molecules diffused by fungi. The Ca^2+^ signal is induced by these diffusible molecules and there appears a biphasic Ca^2+^ trace, which is characterized by a sudden surge in (Ca^2+^) cyt and a small transitionary increase thereafter, that dissipates in about 30 minutes. This Ca^2+^ response is essential for the formation of a polarized cytoplasmic assembly called the perpetration apparatus (PPA), which is required for successful colonization of the plant root by the fungi.

Rhizobial symbiosis is a type of plant-microbe interaction, where legumes establish a mutually beneficial relationship with soil bacteria for fixation of atmospheric nitrogen. Calcium signaling is crucial for this symbiosis as it regulates several key events such as the early stages of root hair deformation, the formation of infection pegs and threads, and the development of nodules. Studies have shown that rhizobia release Nod factors, which trigger the activation of host plant calcium channels, resulting in a rapid influx of Ca^2+^ into the cytosol. This surge in cytoplasmic calcium concentration ([Ca^2+^]cyt) leads to downstream events that promote root hair deformation and initiate infection thread formation (Kosuta et al., 2008). Furthermore, during the later stages of symbiosis, calcium signaling is also involved in nodule development. Research has demonstrated that the activity of the calcium-dependent protein kinase (CDPK) is necessary for the differentiation and proper functioning of nodule cells (Singh et al., 2014). In addition, the process of symbiotic nitrogen fixation is also driven by calcium signaling as it regulates the synthesis of key protein leghaemoglobin (Wang et al., 2016). Calcium-based fertilizers have shown great potential in enhancing symbiotic interactions in plants. The addition of calcium to soil has been reported to enhance nodulation and nitrogen fixation in leguminous crops (Santachiara et al., 2019). The topical application of calcium also enhances the growth of arbuscular mycorrhizal fungi (AMF) and improves the development of AMF structures in roots (Harrison, 2005). This is because calcium ions play a crucial role in the establishment of symbiotic interactions by regulating the signaling pathways involved in the recognition and response of plants to symbiotic microorganisms (Bapaume and Reinhardt, 2012). Studies have shown that calcium-based fertilizers can significantly increase the colonization of plant roots by AMF. For example, Bao et al. (2022) reported that the application of a calcium-based fertilizer improved the colonization rate of maize roots by AMF by up to 40%. Similarly, Berruti et al. (2016) observed that the enrichment of soil with calcium increased the spore density of AMF and improved the growth and yield of tomato plants. Furthermore, calcium-based fertilizers have known to enhance the nodulation and nitrogen fixation in legume crops. For instance, Nakei et al., 2022 demonstrated that the application of calcium nitrate increased the nodulation, nitrogenase activity, and yield of soybean plants. Similarly, Garg and Singh (2018) reported that the application of calcium improved the nodulation, nitrogenase activity, and yield of chickpea plants.

## Novel techniques for studying calcium signaling in plants and microbes

The change in Ca^2+^ signals in the cytosol of plant cells could be measured using Ca^2+^ radioisotopes, Ca^2+^‐sensitive dyes, and other electrophysiological means (Atkinson et al., 1996; Zimmermann et al., 1997). However, recent advances in the study of calcium signaling in plant-microbe interactions offer a more detailed understanding of the complex molecular mechanisms involved. The promising tools and approaches that are currently being used are as follows:

### Live-cell imaging and fluorescent probes

These are powerful tools for studying calcium signaling in real-time. By using fluorescent probes that specifically bind calcium ions, researchers can visualize changes in calcium concentration within live plant cells during interactions with microbes. This technique has been used to monitor calcium changes in plant cells in response to pathogens, hormonal cues, and environmental stressors. Several calcium indicators have been developed, including aequorin, cameleon, and GCaMP, which have different characteristics in terms of sensitivity, kinetics, and spectral properties.

Calcium imaging studies have helped to explore the calcium signaling in diverse plant and microbial systems, including *Arabidopsis*, rice, tobacco, and yeast. Aequorin, one of the earliest developed calcium indicators, which demonstrated that various stimuli can trigger Ca^2+^ signals in plant cells, including biotic stimuli such as yeast preparations, fungal cell wall components, and bacterial pathogens. Aequorin based calcium signaling has been studied in *Arabidopsis and* rice plants (Mithöfer et al., 1999). Ca^2+^ fluctuations have been linked to ROS-mediated defense signaling pathways, and sustained high Ca^2+^ concentration responses have been associated with phytoalexin production. Herbivore-associated molecular patterns (HAMPs) such as volicitin or linolenoyl-L-glutamine have been demonstrated to cause changes in cytosolic Ca^2+^ levels. Studies with lima bean leaves and soybean cell cultures have demonstrated that Ca^2+^ signaling is activated by *Spodoptera littoralis* larvae bites and certain components present in their regurgitate, like linolenoyl-L-Glutamine and volicitin (Maffei et al., 2004). These Ca^2+^ transients are associated with early membrane depolarization and/or hyperpolarization, which strengthens plant defense through systemin, ROS synthesis, and jasmonic acid signaling. Therefore, the regulatory function of Ca^2+^ signaling is highlighted in plant defense mechanisms, where different stimuli can elicit distinct Ca^2+^ variations, exhibited by their form, amplitude, frequency, duration, spatial localization, and the involvement of calcium ion pool, which are all dependent on the type of stimulus perceived by plant cell. Studies on guard cells further confirmed this concept, where the manipulation of Ca^2+^ oscillations can control the stomatal opening and closure for a long term.

The GECIs have been instrumental in studying the real‐time kinetics of Ca^2+^ surge in plant tissues, as a consequence of pathogenic infection or elicitor treatment. Aequorin (AEQ) is a luminescent calcium-sensitive protein, isolated from the bioluminescent crystal jelly, *Aequorea victoria* and is the pioneering GECI used to study Ca^2+^ signaling in plants. The AEQ luminesces upon binding with Ca^2+^ ions and thereby serves as a reporter for any change in Ca^2+^ ions concentration, brought about by touch, cold stress or pathogenic stress (Knight et al., 1991). Miyawaki et al. (1997) pioneered to construct a Ca^2+^ dependent fluorescent indicators that are genetically encoded and can be targeted for specific sub-cellular locations. The GECI cameleon, an engineered protein created for studying Ca^2+^ movement in live cells has been used to study calcium signaling in plants. The Ca^2+^ signature concept was developed through research on *Medicago truncatula* plants expressing the Ca^2+^ probe cameleon YC2.1. A link between Ca^2+^ oscillations induced by bacterial Nod factor and corresponding activation of selected nodulation marker genes by modulating Ca^2+^ homeostasis has been demonstrated pharmacologically. For instance, the ENOD11 nodulin gene required about 30 consecutive Ca^2+^ spikes for induction. Wang et al. (2019), used the fluorescent calcium sensor Cameleon 3.60 to study the dynamics of calcium signaling during *P. indica* and *Arabidopsis* roots interaction. They found that calcium signaling was critical for the colonization of roots by *P. indica*, and that calcium oscillations occurred in a specific temporal pattern during the interaction. Yet another GECI, GCaMP, has been studied for calcium signaling in yeast (Wu et al., 2022). Tang et al. (2017), used the genetically-encoded calcium sensor GCaMP6s to study calcium dynamics during the interaction between the pathogenic bacterium *Ralstonia solanacearum* and tomato plants. They found that calcium signaling activated defense responses in tomato plants and that the bacterial effector protein RipAC disrupted calcium signaling to suppress plant defense responses. Calcium imaging has also been used to study calcium signaling in response to various biotic and abiotic stresses in plants. For instance, calcium imaging has been used to study calcium signaling in response to pathogen infection in *Arabidopsis* (Mithöfer et al., 1999) and rice (Zhang et al., 2015). Calcium imaging has also been used to study calcium signaling during drought and salt stress in *Arabidopsis* (Bartels and Sunkar, 2005; Du et al., 2018). By providing real-time visualization of calcium dynamics, live-cell imaging and fluorescent probes can provide significant insights to explore the molecular mechanisms involved in plant-microbe interactions.

### Genome editing

The CRISPR Cas9 techniques have allowed researchers to directly and objectively monitor the complex interactions between stress signaling and response in plants. As a result, many new connections have been uncovered and many lacunae have been bridged in the complex mechanism of stress tolerance by plants. Crop improvement has been accelerated over the past three decades through genome editing methods that modulate gene activity. The Ca^2+^/CaM-binding proteins have been targeted to generate stress-tolerant crop lines by generating knockout mutation of CML 24 (Ma et al., 2008). While the specific stress-responsiveness of individual Ca^2+^/CaM-binding partners is unclear, CDPK/CIPK regulations have been linked to several abiotic and biotic factors, likely due to their upstream position in the signaling cascade (Pan et al., 2018). Additionally, CAMTAs regulate downstream genes of signaling cascade to mitigate the deleterious effects of various stressors (Doherty et al., 2009; Rahman et al., 2016). CRISPR-Cas9 was used to develop disease-resistant wheat varieties. They identified TaCIPK14, a wheat CIPK homologue gene, and found that TaCIPK14 knockout mutants have higher tolerance to wheat stripe rust caused by *Puccinia striiformis* f. sp. tritici (*Pst).* Furthermore, the researchers generated Tacipk14 mutant plants via CRISPR/Cas9 technology that showed higher resistance to *Pst* without any significant difference in crop yield in comparison to control plants. These results demonstrate the potential of using CRISPR/Cas9 technology to develop disease-resistant wheat varieties by targeting the genes involved in various calcium signatures (He et al., 2023).

Virus-induced gene silencing (VIGS) using Barley stripe mosaic virus (BSMV) has been employed to study the function of various genes, including those encoding Cyclic nucleotide-gated channels (CNGCs) during plant-pathogen interactions. Kang et al. (2019) used BSMV-VIGS to identify TaCNGC14 and TaCNGC16 gene interactions during stipe-rust infection. This BSMV-VIGS has been used to investigate the role of CNGCs in alleviation of biotic and abiotic stresses (Guo et al., 2018; Tian et al., 2019). The significance of CNGCs in plant defense against pathogens has been studied using the gene silencing approach. For instance, Saand et al. (2015) employed BSMV-VIGS to silence CaM2 and CaM6 genes, which implicate SlCNGC17 and SlCNGC18 genes for resistance to tomato against *P. aphanidermatum*, a soil-borne fungal pathogen that causes root rot in tomato. These studies demonstrate the versatility of the BSMV-VIGS approach in studying the functional aspects of CNGCs for their potential role in pathogenic defense. Therefore, identifying a master switch for Ca^2+^ signaling is a high priority for the scientific community to address the challenges posed by biotic stressors.

### Multi omics approaches

The use of innovative “omics” techniques, combined with remarkable advancements in modern molecular biology, has empowered scientists to uncover the intricate interactions between stress signaling and response in plants. Genome-wide studies have helped to identify genes encoding Ca^2+^ signaling elements, which have been extensively studied in plants. The diversity of Ca^2+^ transport elements in plants reflects their evolutionary origins, functional diversification, and roles in stress response. In order to fulfil the intricate and myriad functions of the Ca^2+^ transport system, different types of Ca^2+^ transport elements are present in plants (Taneja and Upadhyay, 2021). *In silico* investigations of genes involved in Ca^2+^ signaling has identified various Ca^2+^ transporters in *Arabidopsis* and rice. Comparative transcriptomic analysis has further revealed the involvement of Ca^2+^ transporters, particularly CNGCs, in mitigation of biotic and abiotic stresses. For example, CNGCs have been implicated in clubroot resistance, sunflower genotype resistance to *Verticillium dahliae*, and rice responses to bacterial pathogens and drought stress (Guo et al., 2017). Also, CNGC genes were implicated in response to *Pseudomonas fuscovaginae* and *Xanthomonas oryzae* pv. oryzae (Xoo), in rice as well as in drought stress in tobacco (Nawaz et al., 2014). Similarly, transcriptomic profiling of *Botryosphaeria dothidea*-infected apple leaves revealed differential expression of MdCNGC genes in response to the pathogen (Zhang et al., 2018). These studies indicate the importance of CNGC genes in plant defense mechanisms against biotic and abiotic stresses and suggest their potential as targets for crop improvement. The transcriptomic profiling has shown that Ca^2+^ signaling elements play crucial roles in regulating the resistance to pathogens in plants. For instance, a study by Tian et al. (2020) used transcriptomic analysis to identify genes that were upregulated in tomato plants infected with the pathogenic bacterium *Ralstonia solanacearum*. The research found that numerous genes involved in calcium signaling were associated with plant defense responses. Previous studies have typically investigated bacteria and host plants separately; however, meta-transcriptomic analysis, provides a more comprehensive information on the simultaneous transcriptional state of a variety of microorganisms. Samaras et al. (2021) performed transcriptomic and metabolomic analysis to investigate changes in cucumber roots after inoculation with *Bacillus subtilis* MBI600, a biocontrol agent. They found that an upregulation of expression of genes involved in plant growth promotion, stress response, and defense-related pathways were upregulated in the treated plants. The metabolomic analysis revealed changes in the levels of metabolites involved in primary and secondary metabolism, including amino acids, organic acids, and phenolic compounds. These results suggest that the *Bacillus subtilis* MBI600-mediated growth promotion and enhanced defense response in cucumber plants may be attributed to the activation of signaling pathways and the biosynthesis of defense-related metabolites. Proteomics studies have also been utilized for identification of proteins involved in calcium signaling pathways in plant-microbe interactions. For instance, a proteomic study discovered calcium-binding proteins and ion transporters as vital components of calcium signaling in the interactions between soybean roots and *Bradyrhizobium japonicum* (Wang et al., 2016).

## Future directions and challenges

The intricate involvement of calcium signaling in plant-microbe interactions presents opportunities for biotechnological applications aimed at enhancing plant growth and improving crop yields. Calcium-based fertilizers have been proposed as potential tools for promoting symbiotic interactions between plants and beneficial microbes, such as endophytes, mycorrhizal fungi and rhizobia. Additionally, the manipulation of calcium signaling pathways in plants through genetic engineering or chemical treatments could be utilized to confer resistance against microbial pathogens or improve stress tolerance.

Despite the potential benefits of harnessing calcium signaling in plant-microbe interactions, there are several challenges that must be overcome. One major hurdle is the complex nature of calcium signaling pathways, which involves a large number of components and regulatory mechanisms. The identification and characterization of key components and their interactions will require further research and development of new techniques for studying calcium signaling dynamics *in vivo*. Another challenge is the specificity of calcium signaling responses, which must be finely tuned to ensure appropriate responses to different microbes and environmental conditions. Understanding the mechanisms of specificity and cross-talk between different signaling pathways will be essential for developing strategies for manipulating calcium signaling in plants.

New research avenues are emerging to provide deep insights into the intricacies of calcium signaling in plant-microbe interactions. For example, advances in imaging techniques and biosensors are enabling the visualization and measurement of calcium signals in real-time, providing a detailed understanding of the spatiotemporal dynamics of calcium connections in plants and microbes. Furthermore, the identification and characterization of novel components of calcium signaling, such as ion channels and transporters, will help to further unravel the complex mechanisms of calcium signaling in plant-microbe interactions. The omics approaches, such as transcriptomics and proteomics, may provide a system-level understanding of calcium signatures in plants and microbes.

## Conclusion

This review highlights the crucial role of calcium signaling in plant-microbe interactions. Calcium signaling pathways in plants involve the release of calcium ions, transporters, and sensors, which play a critical role in plant responses to microbial pathogens and beneficial microbes. The modulation of calcium signaling by microbial effectors, particularly in pathogenic interactions, has been extensively studied. Additionally, calcium signaling has been identified as a crucial mediator in plant-microbe symbiosis, particularly in the establishment of arbuscular mycorrhizal and rhizobia interactions. Since a large number of Ca^2+^ binding and Ca^2+^ decoding proteins are involved in the process of Ca^2+^ dependent signaling, mutation studies for identification of genes responsible for Ca^2+^ surge is recommended as a promising method to unravel this complexity. The study of calcium signaling mechanisms in plant-microbe interactions has significant implications for biotechnological applications, particularly in the development of calcium-based fertilizers to enhance symbiotic interactions. However, there are still key challenges to overcome in understanding the intricacies of calcium signaling in plant-microbe interactions, particularly in the development of novel techniques for studying calcium signaling in plants and microbes. The precise manipulation of Ca^2+^ signals may be harnessed to obtain climate smart crops with enhanced stress tolerance. The generation and dissipation of Ca^2+^ could be controlled using reverse genetic approaches. Overall, the unravelling of calcium signaling mechanisms in plant-microbe interactions provides exciting research avenues for future investigations.

## Author contributions

NN and AR conceived and conceptualized the idea. NN, AR, PN, RP, GP, BC, and DK contributed to writing, editing, reviewing and finalizing the article. All authors contributed to the article and approved the submitted version.
